# Branding water

**DOI:** 10.1016/j.watres.2014.03.056

**Published:** 2014-06-15

**Authors:** Sara Dolnicar, Anna Hurlimann, Bettina Grün

**Affiliations:** a The University of Queensland, Brisbane, Australia; b The University of Melbourne, Melbourne, Australia; c Johannes Kepler Universität Linz, Linz, Austria

**Keywords:** Public acceptance, Branding water, Positioning water, Perceptions of water, Attitudes towards water, Communicating about water

## Abstract

Branding is a key strategy widely used in commercial marketing to make products more attractive to consumers. With the exception of bottled water, branding has largely not been adopted in the water context although public acceptance is critical to the implementation of water augmentation projects. Based on responses from 6247 study participants collected between 2009 and 2012, this study shows that (1) different kinds of water – specifically recycled water, desalinated water, tap water and rainwater from personal rainwater tanks – are each perceived very differently by the public, (2) external events out of the control of water managers, such as serious droughts or floods, had a minimal effect on people's perceptions of water, (3) perceptions of water were stable over time, and (4) certain water attributes are anticipated to be more effective to use in public communication campaigns aiming at increasing public acceptance for drinking purposes. The results from this study can be used by a diverse range of water stakeholders to increase public acceptance and adoption of water from alternative sources.

## Highlights

• Different sources of water are perceived very differently by the public.
• Droughts or floods had a minimal effect on people's perceptions of water.
• Perceptions of water were stable over time.
• Certain water attributes are anticipated to be more effective in public communication campaigns.

## Graphical abstract

**Figure:**
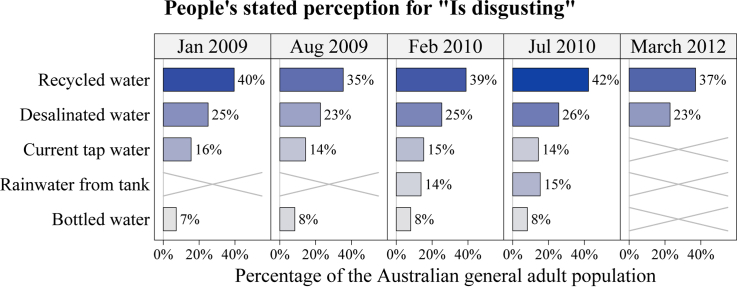

## 1 Introduction

In theory, the problem of water supply shortage is solved: a range of engineering solutions exist which can augment existing water supplies using wastewater, seawater, or water from difficult to procure locations. However, these engineering solutions are insufficient alone to ensure successful implementation. Consideration is needed of the often significant economic, social and environmental costs of such water augmentation projects. In many instances public opposition (perceived or real) to alternative water sources has prevented the implementation of alternative water sources. This opposition can be based on many components including philosophic opposition to augmentation rather than demand management, concern for the siting of such infrastructure, and opposition to the use (particularly potable use) of the alternative water source.

Public support or rejection of alternative water sources is influenced by people's images of different sources of water. Many practical cases are known where people's negative image of recycled water led to the abandonment of plans for such projects, which were to be critical components of the future water supply of the respective regions. Negative images can be actively reinforced by people opposed to water augmentation projects. For example, a community group opposed to the development of a potable water recycling plant in Toowoomba (Australia) heavily communicated what they perceived to be the dangers of recycled water in a successful attempt to prevent the construction of a recycling plant at a public referendum (van Vuuren, 2009a,b; Dolnicar and Hurlimann, 2010; Price et al., 2012).

The case of Toowoomba demonstrates that the image of water matters. The importance of image is well understood in commercial market research, where billions of dollars are spent each year trying to understand brand images of products and developing advertising campaigns to modify or reinforce brand images. Branding is successfully used in the bottled water market, where over 200 billion litres of bottled water were sold worldwide in 2008 (Gleick and Cooley, 2011). Wilk (2006) argues that cultural branding has been successful in turning water into a consumer good. Despite having a clean, cheap and safe supply of water delivered to their homes, many people in developed nations are willing to spend significant amounts of money buying bottled water (Wilk, 2006). This is in contrast to several cities in developing nations where demand for bottled water is driven by the fact that centralised supplies, if provided at all, fail to meet basic criteria for drinking water quality (UNESCO, 2006).

Despite the importance of water to supporting human life, the image of water has not been extensively studied (one exception is the study by Dolnicar and Schäfer (2009) which reports – based on a one-off cross sectional survey study – on perceptions the Australian population holds about four kinds of water: recycled water, desalinated water, tap water and bottled water). What is lacking is knowledge of the images people hold of a range of water sources, how these images differ between sources, and across a comprehensive range of potentially perceived water attributes. Additionally, knowledge relating to how these perceptions may vary over time and in relation to significant water events is limited.

The reason for the lack of study of water images may be that water is predominantly supplied to consumers in cities of developed nations in a centralised monopoly commodity situation. Thus, there may be little need for public policy makers or water companies to invest in understanding the public image of water and developing branding and positioning approaches to improve the image of a specific type of water. Or, if they do conduct such studies, they may not be making them publicly available. There are limited examples of branding campaigns conducted by authorities responsible for centralised water supplies. Examples include “Tap™” (Sydney Water, 2014) which highlights the environmental benefits of tap water, and asks members of the public to ‘pledge’ to drink tap. Another notable example is the marketing of NEWater in Singapore – with the introduction of recycled water into the nation's supply, including for drinking purposes (PUB, 2014). This was associated with the distribution of bottles of NEWater to the public when launched, and a visitor centre. The majority of such examples provide little publically available information of the factors motivating these activities, of the research undertaken to inform them, or of any critical analysis of their success or otherwise.

The lack of publically available information about the image of drinking water means its image is not well understood, and there is little on which to base systematic communication with people to either reinforce (positive) or modify (negative) images. Additionally, it means there is limited information on which to base decisions and communications regarding the use of alternative water sources, which has and will continue to be an increasing imperative in the future, given the predicted impacts of climate change on water resources in many locations across the globe (Bates et al., 2008).

The present study builds on the work by Dolnicar and Schäfer (2009) and investigates the following research questions: Which attributes of water are seen by the public as desirable and undesirable (Research Question #1)? What image does the public have of different water sources (specifically tap water, bottled water, recycled water, desalinated water, and water from one's own rainwater tank), and are these images different from one another (Research Question #2)? Do water images remain stable over time (Research Question #3)? Which water attributes are most powerful for branding or (re)positioning campaigns (Research Question #4)?

Throughout this paper Keller's (1993, p. 2) definition of the term “image” is adopted: “the set of associations linked to the brand that consumers hold in memory”. The term “brand” is used to refer to the different sources of water studied.

The study is based in Australia, which allows for an interesting case study of water. Major cities have traditionally been supplied water through centralised supply systems aided by dams to capture rain runoff and conveyed to the population through pipes (Dingle and Rasmussen, 1991). Locations across the country have periodically experienced drought, most recently for many major urban settlements in the country during the 2000s. For many of these locations, the drought ended with devastating floods. As a consequence, water was a major topic of public debate and most states initiated water augmentation projects to secure future water supply given the projected shortfall between demand and supply.

Findings from this study can be used by water authorities, public policy makers and water retailers to develop and maintain more positive water brand images.

## 2 Sources of water

The source of water which a population draws upon for consumptive use differs across the globe, depending on a location's physical and geological characteristics and the consideration of economic and environmental efficiency. However, the water source used can change over time, influenced by change to factors such as environmental and climatic conditions, population size and economic circumstances. These are important considerations, because an ample supply of water has historically been a key determination of a population's ability to grow (Mumford, 1989).

In developed nations, water supplies predominantly take the form of centralised systems. In many locations, water has traditionally been drawn from surface and ground water storages (World Resources Institute, 2002). Until recently, energy intensive sources of water – such as seawater desalination – were limited to arid countries largely located in the Middle East (Lattemann et al., 2010), and planned potable reuse seldom occurred. However, the use of alternative water sources such as desalinated seawater and the planned use of recycled water to augment traditional supplies has rapidly increased since the 1990s due to the decreasing cost of technology, the increasing cost of freshwater treatment and marginal water source removal (Lattemann et al., 2010), and the increasing total demand for water.

In many locations there is not simply one source of water, but a suite of sources drawn upon to meet demand. The exact environmental and economic cost of each source of water varies depending on a location's physical characteristics. However, some alternative sources of water, such as desalination, have been acknowledged to have high environmental and economic impacts due to treatment processes and by-products, and high energy use (Morton et al., 1996; Schiffler, 2004). Other sources such as recycled water, have given rise to significant public and institutional opposition (Committee on the Assessment of Water Reuse as an Approach to Meeting Future Water Supply Needs and National Research Council, 2012; Hurlimann and Dolnicar, 2010).

However, in developing nations, centralisation is not as wide spread, and the reliability of such systems (when they do exist) is poor at times. Many households in such settings seek alternative sources of water for reasons of availability, shortage, negative pressure, contamination and unplanned settlement patterns (Dutta et al., 2005; Pattanayak et al., 2005). In such circumstances, perceptions about poor quality of centralised supplies have led some consumers to boil water, buy bottled water or install filters (Um et al., 2002). More recently – in countries such as Australia – substitution with alternative water sources has been found to occur with a significant proportion of the population, driven by water shortage and restrictions. Hurlimann (2011) found that, in 2008, 74 per cent of the Victorian population connected to a centralised water supply sometimes or always used an alternative source of water for the purpose of garden watering. Specifically, 25 per cent substituted rainwater from personal tanks for garden watering, 12 per cent for car washing, and 9 per cent for drinking. The context outlined above indicates that water sources drawn upon by utilities are likely to change in the future, yet there is little information for utilities and public officials to draw upon with regards to understanding public responses to these changes.

In the Australian context a number of specific factors need to be considered: in 2010/11 the predominant source of water for consumptive purposes was surface water (92 per cent), providing 6,532 GL, followed by ground water with 454 GL. Recycled water provided 351 GL, and desalination plants provided 121 GL (ABS, 2012). The use of recycled water and desalinated water had increased since the previous water account; however their overall consumption remains a small fraction of the nation's total (ABS, 2012).

In Australia, The Australian Drinking Water Guidelines (NHMRC and NRMMC, 2013) define “safe, good quality water, how it can be achieved and how it can be assured” (p.1) from both a public safety and aesthetic quality standpoint. These guidelines apply to all sources of water intended for drinking except bottled or packaged water, which are subject instead to the *Food Standards Code* (Food Standards Australia New Zealand, 2011). The consumption of bottled water has a long history, but its use in countries with a safe supply of centralised drinking water is filled with controversy (Gleick and Cooley, 2009; Parag and Roberts, 2009). While the industry enjoyed a period of strong growth, this slowed a little, and is said to be attributable to factors in the USA, including the slowing economy and increasing awareness of environmental impacts of bottled water (Hein, 2008).

Rainwater from personal tanks is used for potable purposes in 13 per cent of households in Australia (Australian Government, 2004). Consumption of rainwater is high in the state of South Australia, where 42 per cent of households use it for drinking (Heyworth et al., 1998), with higher use in rural areas compared to urban. This high use of rainwater is attributed to poor aesthetic quality of mains water and fear of chemical content (Heyworth et al., 1998), hence demonstrating the importance of water image. However, as noted in the Australian Government's (2004) *Guidance on the use of Rainwater Tanks*, the general public perceive rainwater is safe to drink. It is also acknowledged in this guidance that while the risk from consuming rainwater is low in most areas of Australia, water from such tanks is not as well managed and treated as the urban supplies. Thus, this represents a potential gap in aesthetic attributes, actual quality, and public image.

Major water supply management incidents can have the potential to impact the public image of water. One such example is the Sydney Water Crisis, where the city's water supply (surface water) was contaminated on several occasions between July and September 1998, resulting in boil water alerts – the case is described in detail by Hrudey and Hrudey (2006). A 40 per cent growth in bottled water sales in the following year was attributed to the crisis (Doria, 2006). A study by Sydney Water conducted in 1995 and 1999, found trust in the water authority to ‘manage recycled water responsibly’ had fallen from 60 per cent in 1995, to 41 per cent in 1999 (Sydney Water, 1999), the year after the incident. Limited publically available research has been conducted on the impact of this incident on the image of Sydney's water supply. On the contrary, Hurd (1993) found that community perceptions and attitudes towards municipal water supply in the USA were relatively stable even after a Cryptosporidium outbreak.

## 3 Prior work on water image

Research into consumer beliefs regarding various aspects of drinking water has a long history. Particular attention has been paid to evaluating aesthetic attributes and threshold values for components of the water at which it becomes unacceptable for drinking: for example, research shows that there is a relationship between beliefs of water quality and actual total dissolved solids levels (ARCWS, 1999; Bruvold, 1968, 1970; Syme and Williams, 1993).

Doria (2010) conducted a comprehensive review of how people assess drinking water quality. Factors that emerged include risk perception; water chemicals and microbiological properties; contextual indicators; prior experience; impersonal and interpersonal information; trust in the water companies and other groups; perceived control; demographics, cultural background and world views. The review was focused on drinking water quality in general, it did not investigate differences across water sources. It could be assumed that beliefs the public holds about different souces of water are influenced by the above factors, in addition to source specific perceptions.

Research has been conducted to understand the reasons people are willing to buy and drink bottled water over water delivered through a central supply. Findings are varied and relate to perceptions surrounding the relative safety of the water source, healthiness, and taste preference, with some people substituting bottled water for soft drinks and other beverages (Hurd, 1993; Mackey et al., 2003). Doria (2006) reviewed academic and grey literature on this matter and found that the main factors attributed to this in consumer surveys were aesthetic attributes, and health/risk concerns. Other contributing factors include demographics, perceived quality of the tap water source, and trust in water companies. Additionally, in a large Australian study, Marks et al. (2006) found that, while most respondents did not perceive a health risk associated with their supply, those that did were very likely to change their source of drinking water.

Research into public acceptance of recycled water also has a long history, but has rapidly intensified over the past decade as interest in recycled water increased internationally. Early work found that people distinguish between purposes of water use, with close to body uses such as drinking being less accepted than public uses such as landscape irrigation (Bruvold, 1972; Bruvold and Ward, 1970). These findings have been confirmed in many studies since (including Marks et al., 2006; Dolnicar and Schäfer, 2009; Lohman and Milliken, 1985). Research has also focused on understanding who is most likely to support the use of recycled water and why, with various demographic and attitudinal factors found to contribute (Hurlimann et al., 2009; Dolnicar et al., 2011).

More recent research has attempted to understand these preferences further. Hurlimann and McKay (2007) investigated an Australian community's preferences for various attributes of recycled water for various uses. Their results indicate that the importance placed on aesthetic attributes varies depending on the use of recycled water. For garden watering, having ‘low salt levels’ was the most important attribute studied, for clothes washing ‘colourless,’ and for toilet flushing a ‘low price.’ At the time of Hurlimann and McKay's study, the community was not were using recycled water. However a follow-up survey was conducted in 2007, when recycled water had been used for a period of time through a dual pipe system. Hurlimann (2009) found that 28 per cent of respondents perceived the recycled water to have an odour, and 49 per cent perceived a colour. This reflects findings by Marks et al. (2002) in New Haven (Adelaide, Australia): users of recycled water – for toilet flushing only – reported an occasional odour, murky colour and the presence of sediment. Only 35 per cent of study participants had connected a tap to the recycled water system. Similarly a Danish study (Albrechtsen, 2002) compared the microbial water quality of seven rainwater systems, four graywater systems and eight traditional systems, reporting several consumer complaints relating to bad smells associated with the graywater systems. In one case this led to the shutdown of the plant.

Few studies have compared beliefs the public holds about different water sources. Most comparisons are limited to the investigation of tap water and bottled water discussed earlier. Additionally, many comparisons focus on likelihood of use, with less work conducted on the exploration of beliefs. In a review of recycled water research, Dolnicar and Saunders (2006) identified the need for research into different sources of water and messages supporting adoption of recycled water including branding research. Such research has been conducted recently, particularly comparing desalinated and recycled water.

Dolnicar and Schäfer (2009) compared Australians' beliefs about recycled, desalinated, tap and bottled water across thirty characteristics concluding that bottled water was perceived as the most irresponsible source of water on environmental terms, followed by desalinated, tap then recycled water. Desalination was acknowledged to use a ‘lot of energy in production,’ followed by bottled, recycled then tap water. With regards to health issues, recycled water was seen as the unhealthiest, followed by desalinated, then tap and bottled water. Tap water was associated with a number of negative characteristics compared to desalinated and bottled water (e.g. was more likely to be perceived as having a colour and odour), hence providing potential marketing advantages for alternative water sources. To the best of the authors' knowledge this was the first and only study to date which has studied beliefs the general population holds about four sources of water. The limitations of this study are that they asked respondents whether they perceived each water source had certain attributes, they did not assess how desirable or undesirable each attribute was. Additionally, the analysis was based on one single cross-sectional data set. These limitations are addressed in the present study, thus moving from a description of water images towards the analysis of ideal water images, which are more useful to water stakeholders in terms of developing promising communication messages.

## 4 Methodology

Data was collected in five cross-sectional online survey studies using nationally representative samples of the adult Australian population in January 2009 (1495 respondents), July 2009 (1750 respondents), January 2010 (1003 respondents), July 2010 (1000 respondents), and March 2012 (999 respondents). Data was collected using professional research-only online panel companies (Research NOW and Survey Sampling International). Respondents registered on the panel were invited to participate in the survey via email and received a compensation of four Australian Dollars for their participation; this amount is in line with the fieldwork companies' standard compensations for survey participation which is dependent on the length of the survey and ranges from $1 to $5. Invitations were sent out to a representative sample of the adult Australian population. The number of invitations sent out was based on the sample size requirement for each wave, typically 1000 validly completed questionnaires, and the known panel response rate of between 15 and 20 percent. In addition, quotas were set to avoid over-representation of certain subsets of the population.

Respondents were asked about their perceived image of various water sources, water-related behaviours, and personal characteristics. Each source of water was assessed by respondents along a set of attributes which were developed in collaboration with water experts and first used in Dolnicar and Schäfer’s (2009) study; the full list of items is shown in Table 3. A complete questionnaire is provided in the online supplementary materials. Survey respondents ticked “yes” if they felt that an attribute applied to a specific source of water or “no” otherwise. This format is known as forced choice binary format or the binary with inferred threshold measure and has been shown to lead to the most reliable results in terms of test-retest reliability in brand image measurement (Dolnicar and Grün, 2013; Dolnicar et al., 2012; Dolnicar and Leisch, 2012; Rossiter, Dolnicar and Grün, in press).

Finally, it should be noted that, during data collection, many locations across Australia were experiencing a very serious drought. In parts of Queensland, Victoria and New South Wales, the drought ended with significant rainfalls in 2011, associated with devastating floods which caused significant loss of property and life. As a consequence, the water situation during the last survey wave in March 2012 was substantially different from that in previous survey stages: by this time the water supply levels in many Australian capital cities had replenished to levels which were no longer of an emergency situation. For example, the total level of Melbourne's dams combined was 33 per cent in January 2009; 27 per cent in July 2009; 36 per cent in January 2010; 36 per cent in July 2010; and 65 per cent in March 2012.

## 5 Results

### 5.1 Sample characteristics

Table 1 provides an overview of the characteristics of the sample across all survey waves for: state of residence, age, and gender. Statistical analysis reveals that there were no significant differences in demographic characteristics across survey waves except for age, which was significantly higher in wave 5 (*χ*^2^ test for gender: *χ*^2^ = 0.33, *df* = 4, *p*-value = 0.99; χ^2^ test for state: *χ*^2^ = 7.1, *df* = 28, *p*-value = 1.00; ANOVA for age: *F* = 5.5, *df*_1_ = 4, *df*_2_ = 6242, *p*-value < 0.001). Gender and state of residence closely matched the ABS profiles, and age was higher – this is reflective of the fact that only adults were sampled, and the survey company was asked to recruit a sample representative of ABS age categories.

Table 2 contains information about a number of variables collected, including: respondents' previous use and self-assessed level of knowledge for each source of water; effort made to learn about water; and water preference for drinking.

### 5.2 Research Question #1: which attributes of water are seen by the public as desirable and undesirable?

Water attributes included in the online survey are provided in Table 3 and are ordered by the percentage of respondents who state that these attributes are desirable to them in the survey data collected in July 2009. Specifically, respondents were asked the following question: “Please indicate for each water attribute listed below whether it is desirable or not for your household water to have this attribute”.

As can be seen, being healthy emerges as most desired attribute, followed by being safe for human consumption, being odourless, looking absolutely clear, being the most responsible source of water from a public health perspective, and water providers being trustworthy. All of these attributes were rated desirable by at least 94 per cent of respondents. Eighty per cent of respondents indicate that they want their water to have all of these six characteristics.

### 5.3 Research Question #2: what images does the public have of different sources of water and are they different from one another?

Data collected in January 2010 was used to provide the benchmark image of different sources of water because it was the first to contain questions about all the sources of water of interest. The images of different sources of water for the survey data from January 2010 are provided in Table 4 for desirable attributes, and in Table 5 for undesirable attributes.

Differences between the average evaluations of the five water sources are significant for each attribute.

### 5.4 Research Question #3: do water images change over time?

To determine whether water images change over time, all five available data sets were analysed. Note that not all water sources were included in all survey waves: for recycled and desalinated water measurements across five points in time are available, for bottled water and tap water, four measurements are available and for rainwater from personal rainwater tanks, only two measurements are available. Changes of water images are shown in Table 6 for desirable attributes and in Table 7 for undesirable attributes. Given the data indicated that a large change or trend in change did not occur, the observed variation in agreement levels was decomposed for each attribute into (1) the variation which can be attributed to the water source, (2) the variation which can be attributed to the survey wave and (3) residual variation. The proportion of variation explained by the water source is in all cases at least 93%, confirming that time has not affected water images much.

Additionally, the variation was decomposed separately for each water type into (1) the variation which can be attributed to the different attributes, (2) the variation which can be attributed to the survey wave and (3) residual variation. Again for each water type the proportion of variation explained by attribute alone is high with at least 94% over all waves available. A specific comparison of the last two waves including only recycled water and desalinated water indicates that the variation due to attribute is 92% for recycled water and 98% for desalinated water.

### 5.5 Research Question #4: which water attributes are most powerful for branding or (re)positioning campaigns?

The importance of attributes was assessed by using the respondents' ranking of the five water types for drinking water preference as the dependent variable. The evaluation of the same water types on the different attributes as well as the water types themselves were used as explanatory variables. Only data from the survey waves collected in January and July 2010 (where all five water types were ranked) were used. The different overall preferences of the five water types were accounted for in the analysis. A binomial logit model was fitted by reformulating the first and second choice as the result of a pair wise comparison, i.e., where the most preferred water type was compared to the second water type. The differences in evaluation between the two water types on the attributes and the water types compared were used as explanatory variables. The relevant attributes for predicting preference for drinking were selected using the LASSO (least angle shrinkage and selection operator) approach (Tibshirani, 1996; Friedman et al., 2010). Then, a standard binomial logit model was fitted using as explanatory variables only the attributes and water types that have a non-zero coefficient in the LASSO model with the “best” penalty. The “best” penalty was selected using cross-validation where the penalty corresponds to the smallest model with a performance within one standard deviation of the model with best performance. As performance criterion binomial deviance was used.

**Fig. 1:**
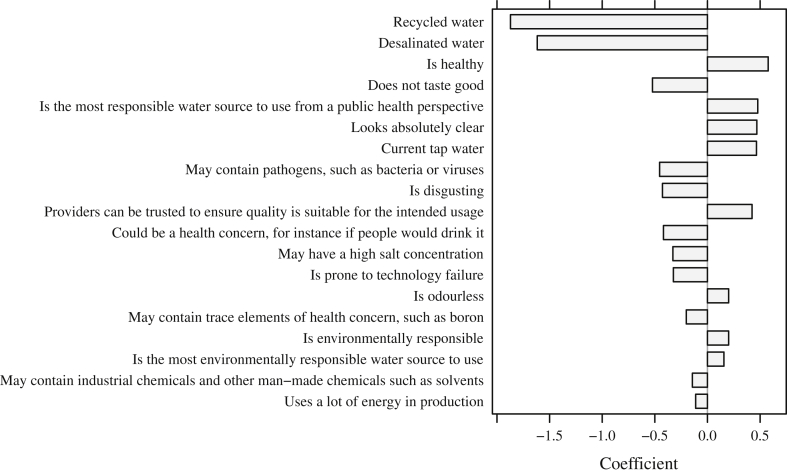
Water attributes influencing willingness to drink.

Fig. 1 contains only the water types and attributes which are strongly associated with people's stated willingness to drink water of a certain kind, i.e., are selected by the LASSO procedure. The bars indicate the extent to which they either positively or negatively influence willingness to drink.

## 6 Discussion

As can be seen in Tables 4 and 5, the brand images of water differ significantly for each attribute. Bottled and tap water are seen to be safe for human consumption and healthy, in contrast to both desalinated and recycled water which were given less positive health ratings. This image of bottled water is interesting, given as discussed earlier is interesting, given that in Australia bottled water is not subject to the same guidelines as drinking water from other sources. Recycled water is perceived as safe for human consumption by the smallest proportion of respondents.

Bottled water performs best on the physical appearance criteria of being absolutely clear and odourless. This image is consistent with previous research which has found that some consumers use bottled water in preference to tap water for aesthetic reasons (Um et al., 2002; Doria, 2006). Rainwater outperforms tap water on absence of odour, and recycled water is perceived as odourless by only 54 per cent of respondents. Rainwater from the tank is perceived as absolutely clear by only 58 per cent of respondents, followed by recycled water (63 per cent).

Tap water and rainwater from tanks are perceived as the most responsible water source in terms of public health. Bottled, desalinated and recycled water are perceived in this way by only about 40 per cent of respondents. This image of rainwater from tanks is important for water managers to understand, given the acknowledged potential for contamination in the Australian Government's (2004) *Guidance on the use of Rainwater Tanks.*

Rainwater from tanks and recycled water are perceived as most environmentally responsible: 90 per cent of Australians believe that rainwater from one's own tank and 54 per cent believe that recycled water is the most environmentally responsible source of water; only 13 per cent believe that bottled water is. This awareness of the environmental impact of bottled water is one of the reasons attributed to a recent decrease in bottled water sales in the USA (Hein, 2008).

Desalinated water is seen by a substantial proportion of respondents as environmentally responsible. This may relate to the low level of knowledge about water reported indicated in Table 2, and in a 2008 Australian study (Dolnicar and Hurlimann, 2009). Approximately 80 per cent of respondents believe that desalinated water, recycled water and rainwater from people's own tanks increase the availability of freshwater. Consistent with these responses, the vast majority of respondents also perceive that those three sources of water have the potential to save Australia from a drought, thus reducing the need for water restrictions. Recycled water is perceived by 63 per cent as reducing contamination of beaches, thus offering a positive side-effect beyond the provision of water.

In terms of undesirable attributes (Table 5), recycled water is perceived by the comparatively largest proportion of respondents as disgusting (39 per cent). Only eight per cent of respondents perceive bottled water as disgusting. Similarly, 52 per cent of respondents perceive recycled water does not taste good, 43 per cent say the same about desalinated water and about one third of respondents each about tap and tank water. Eighteen per cent of respondents dislike the taste of bottled water. Previous research has found that preference for water source is influenced by experience – for example the tap water in a location which someone has grown up in is preferred to other sources of water (see Doria, 2010 for a discussion).

In terms of a range of health concerns (containing trace elements, industrial chemicals, hormones, human waste), recycled water is consistently perceived as performing worst, followed by desalinated water, tap water, rainwater and bottled water. Only with respect to containing pathogens respondents perceive another source of water as more susceptible of containing them: rainwater from a tank. Not surprisingly, therefore, recycled water is most frequently, by 60 per cent of respondents, perceived as a potential health concern if used for drinking. Forty five per cent of respondents share this concern for rainwater, 36 per cent for desalinated water and 21 per cent for tap water.

Concerns about high levels of salt concentration are expressed most frequently with respect to desalinated water (52 per cent of respondents). Recycled water is perceived as staining the washing by more respondents than is the case for other sources of water. This concern about the colour of recycled water is consistent with prior research (Hurlimann and McKay, 2007; Hurlimann, 2009).

Finally, in terms of the cost of provision of the different sources of water, 90 per cent of respondents perceive bottled water as expensive, 82 per cent perceive desalinated water to be expensive, 63 per cent recycled water, 38 per cent tap water and only nine per cent water from a rainwater tank.

It can be concluded from these results that residents' images of different sources of water differ significantly and systematically with recycled water being associated most with potential health issues, bottled water and desalinated water with high prices and low environmental responsibility, and rainwater as cheap and most environmentally friendly.

From the results presented in Tables 6 and 7 and associated statistical analysis, it has to be concluded that water images have not changed substantially over the study period. This is despite the fact that during this time Australia experienced the end of a serious decade-long drought which was accompanied by extensive public debate about water augmentation options to secure Australia's future water supply and drought-breaking devastating floods in 2011. This change of water circumstance was reflected in survey wave 5, but did not appear to have affected the image Australians' have of recycled and desalinated water. As previously discussed, Hurd (1993) found stability of community perceptions and attitudes towards municipal water supply in the USA after a Cryptosporidium outbreak.

Fig. 1 shows which of the desirable and undesirable attributes of water best predict whether or not people express their willingness to drink it. This analysis is of particular importance as it points out to water managers which attributes are most important and thus should be discussed in public information campaigns. The information can also be utilised if positioning and rebranding action is taken.

Results provided in Fig. 1 indicate that regardless of their brand image evaluations, recycled and desalinated water are less likely to be preferred for drinking, whereas current tap water has a higher likelihood to be the preferred water source for drinking. The attributes of safety for human consumption, being healthy, looking clear, and responsible in terms of public health, are the most influential attributes. On the negative side, influential attributes include: not tasting good, containing pathogens, appearing disgusting, being a health concern if people would drink it, being prone to technology failure, having a high salt concentration, containing trace elements of health concern, and containing chemicals and using a lot of energy in production.

Overall, findings resulting from this study add to the limited body of work on attributes people associate with different kinds of water (ARCWS, 1999; Bruvold, 1968, 1970; Dolnicar and Schäfer, 2009; Doria, 2010; Hurd, 1993; Hurlimann and McKay, 2007; Mackey et al., 2003; Syme and Williams, 1993). The following key insights emerge: (1) the public has a robust collective perception of which water attributes are desirable and undesirable, (2) the images of different water sources along those attributes differ significantly, (3) the images of different sources of water are stable over time, (4) despite major external changes specifically a major drought phase and the breaking of the drought leading to serious flooding events in many regions in Australia, the images of desalinated and recycled water were stable over time. Finally, (5) a list of attributes which can be used for rebranding exercises of water has been identified, including both attributes which significantly increase people's stated willingness to drink it and attributes which significantly decrease this willingness.

These findings have major practical implications for public policy makers and developers of water augmentation projects. Firstly, building on the findings of Dolnicar and Schäfer (2009), it is important to recognise the distinctly different images held by the public with respect to different sources of water. Such insight enables water managers and public policy makers to identify the key positive attributes that can be reinforced, and key negative attributes that need to be addressed specifically in public consultation or information processes. This complements existing research which indicates the importance of effective communication (Hurlimann, 2008; Khan and Gerrard, 2006), by suggesting positive and negative communication messages.

The present study has revealed a number of image attributes which can proactively be used to argue, in a positive way, in favour of the development of water augmentation projects (for example, recycled water reduces the need for water restrictions, reduces the contamination of beaches, reduces the amount of wastewater discharged to the environment and creates new jobs). At the same time negative attributes have been identified (e.g. that recycled and desalinated water is disgusting, tastes bad, stains washing, contains salt; and health concerns related to all sources of water, but mostly recycled water) which, in the opinion of the authors, cannot be resolved through advertising because they require the public to have a certain level of understanding of how the water is produced. In such cases, a combination of measures is advisable, including information provision (including information on which countries in the world already use these sources of water and have done so without any incidents for many years), opportunities for the public to visit water augmentation plants, opportunities for the public to experience first-hand the sources of water and extensive public consultation. These have been identified as necessary components by other scholars (including: Dishman et al., 1989; Hurlimann, 2008; Khan and Gerrard, 2006; Law, 2003).

The comparative data provided in this study is particularly useful for the development of public information and consultation because it reveals clearly that the currently dominant form of water in Australia (tap water originating from dams and purified to a high standard) is not seen as the perfect source of water: for example, it is seen by 46 per cent as prone to technology failures (which may be due to incidents with tap water contamination in Australia, most notably in Sydney, see Hrudey and Hrudey, 2006) and 34 per cent state it does not taste good.

Another important finding emerging from this study is that water images in Australia did not change substantially over the period January 2009–March 2012, despite major events, such as droughts and floods. From a public policy perspective this is both an encouraging and discouraging finding. It is discouraging that people appear not to have adjusted their negative images of some sources of water in times where water was so limited that large scale water augmentation in future appeared unavoidable. On the other hand, the sudden availability of water did not lead to the rejection of water alternatives which people saw as viable alternatives before the end of the drought. The findings of the high level of image stability of different sources of water by the general public further highlights the importance of proactively managing water images though a range of channels, because it cannot be assumed that random external events will lead to major attitude changes.

The study has a few limitations: the data was collected in Australia only. Australia is an interesting country to study because of its unique water context, and the relatively recent introduction of water augmentation projects. It is likely, however, that countries which have been reusing or desalinating water over a longer period of time will hold different water images. Furthermore, respondents were asked to assess different sources of water in different survey waves. Optimally, measurements for all attributes and all kinds of water would be available for analysis. Finally, stated intentions of use were used as the dependent variable.

Future work of this nature collecting data internationally would be extremely interesting as it would allow insight into whether water images reflect local water circumstances or whether they remain stable, as they did in Australia through times of dramatic change in the water circumstances. Most importantly, however, it would be beneficial to replicate the study using actual behavioural dependent variables, rather than reported intention to use water from different sources for different purposes.

## 7 Conclusions

The study, based on surveys with 6247 respondents undertaken between 2009 and 2012, leads to the following key insights:

(1) different sources of water – specifically recycled water, desalinated water, tap water from centralized supply and rainwater from personal rainwater tanks – were each perceived very differently by the public,
(2) external effects, which are out of the control of water managers', such as droughts or floods, affected people's perceptions of water to only a small extent,
(3) perceptions of water held by the general public were stable over time, and, most importantly,
(4) certain attributes of water are anticipated to be more effective to use in public communication campaigns in order to increase public acceptance of particular water sources.

## Figures and Tables

**Table 1:** Sample characteristics.

		Wave 1	Wave 2	Wave 3	Wave 4	Wave 5	Aggregate	ABS^a^
Period		2009–01	2009–07	2010–01	2010–07	2012–03		2013
Sample size		1495	1750	1003	1000	999	6247	
Age (in years)	Mean	43.7	43.5	43.9	42.7	45.8	43.9	38
	Standard dev.	15.8	15.6	15.5	15.2	15.6	15.6	
Gender	Male	50.4%	49.7%	49.3%	50.0%	50.3%	50%	50%
State	New South Wales	32.6%	32.9%	33.0%	33.2%	31.5%	33%	32%
	Victoria	25.4%	24.9%	25.2%	24.7%	26.2%	25%	25%
	Queensland	20.0%	20.0%	19.4%	19.3%	19.2%	20%	20%
	South Australia	8.2%	8.0%	8.2%	8.6%	8.3%	8%	8%
	Western Australia	9.5%	10.1%	10.0%	10.2%	10.0%	10%	10%
	Tasmania	2.0%	2.0%	2.1%	2.3%	2.0%	2%	2%
	Northern Territory	1.1%	1.0%	0.9%	0.6%	0.9%	1%	1%
	Australian Capital Territory	1.3%	1.1%	1.2%	1.1%	1.8%	1%	1%

a 2013 data sourced from the Australian Bureau of Statistics (ABS, 2013).

**Table 2:** Respondent experience, knowledge and preference for various water sources.

			Wave 1	Wave 2	Wave 3	Wave 4	Wave 5
% Prior knowledge with …	Desalinated water	No	87%	87%	67%	68%	60%
		Not sure			15%	16%	22%
		Yes	13%	13%	18%	16%	18%
	Recycled water	No	65%	64%	53%	54%	47%
		Not sure			17%	19%	24%
		Yes	35%	36%	30%	26%	30%
	Rainwater from tank	No			13%	13%	
		Not sure			1%	2%	
		Yes			85%	84%	
% Who state they have made a … effort to learn about water		Absolutely no effort	18%	16%			
		A small effort	58%	61%			
		A big effort	21%	20%			
		A huge effort	3%	3%			
% Who state that they know a lot about …	Bottled water				49%	51%	
	Current tap water				49%	51%	
	Desalinated water				31%	34%	36%
	Recycled water				33%	32%	36%
	Rainwater from tank				50%	50%	
First preference	Bottled water				28%	27%	
	Current tap water				45%	44%	
	Desalinated water				1%	3%	
	Recycled water				1%	1%	
	Rainwater from tank				24%	26%	

**Table 3:** Water attributes and desirability levels in July 2009.

	% Respondents who view this attribute as desirable
Is healthy	96%
Is safe for human consumption	95%
Is odourless	95%
Is the most responsible water source to use from a public health perspective	94%
Looks absolutely clear	94%
Providers can be trusted to ensure quality is suitable for the intended usage	94%
Is environmentally responsible	92%
Increases the availability of freshwater	91%
Is the most environmentally responsible water source to use	90%
Can save Australia from drought	90%
Reduces contamination of beaches	87%
Using it reduces the amount of wastewater discharged to the environment	84%
Creates new jobs	84%
Reduces the need for water restrictions	82%
May contain purified domestic wastewater	36%
Contains chemicals, such as chlorine	34%
Requires chemicals to be produced	25%
Quality can be affected by the way it is transported to your home	24%
Producing it could be an environmental concern	22%
May contain purified industrial wastewater	21%
Produces greenhouse gas emissions	19%
Is expensive for the consumer	17%
Is prone to technology failure	16%
Is expensive to produce	15%
Could be a health concern, for instance if people would drink it	15%
Uses a lot of energy in production	15%
May contain pathogens, such as bacteria or viruses	15%
Is expensive to be delivered to the consumer	14%
Because the water cycle is closed, it contains human waste	13%
May contain substances such as hormones, etc., which can affect human fertility	13%
Does not taste good	12%
May contain industrial chemicals and other man-made chemicals such as solvents	10%
May contain trace elements of health concern, such as boron	10%
May have a high salt concentration	9%
Stains the washing	8%
Is disgusting	7%

**Table 4:** Perceptions of water by water source – desirable attributes for January 2010.

	Bottled water	Current tap water	Desal. Water	Recycled water	Rainwater from tank	Chi-square statistic	Deg. of freedom	*p*-value
Is safe for human consumption	93%	90%	74%	54%	69%	559.1	4	<0.001
Looks absolutely clear	94%	71%	73%	63%	58%	361.3	4	<0.001
Is odourless	87%	61%	62%	54%	69%	284.9	4	<0.001
Is healthy	82%	75%	58%	44%	67%	379.0	4	<0.001
Is environmentally responsible	25%	64%	56%	84%	92%	1209.7	4	<0.001
Increases the availability of freshwater	41%	38%	79%	79%	83%	882.2	4	<0.001
Providers can be trusted to ensure quality is suitable for the intended usage	69%	69%	60%	53%	67%	84.8	4	<0.001
Creates new jobs	63%	34%	90%	88%	35%	1262.2	4	<0.001
Can save Australia from drought	23%	28%	77%	83%	79%	1482.2	4	<0.001
Reduces the need for water restrictions	23%	23%	77%	83%	84%	1679.9	4	<0.001
Using it reduces the amount of wastewater discharged to the environment	28%	32%	43%	84%	68%	943.2	4	<0.001
Is the most responsible water source to use from a public health perspective	43%	66%	38%	35%	62%	311.0	4	<0.001
Is the most environmentally responsible water source to use	13%	40%	31%	54%	90%	1375.6	4	<0.001
Reduces contamination of beaches	24%	30%	37%	63%	54%	445.0	4	<0.001

**Table 5:** Perceptions of water by water source – undesirable attributes for January 2010.

	Bottled water	Current tap water	Desal. water	Recycled water	Rainwater from tank	Chi-square statistic	Deg. of freedom	*p-value*
Is expensive to be delivered to the consumer	90%	38%	82%	63%	9%	1811.2	4	<0.001
Uses a lot of energy in production	77%	34%	91%	72%	7%	1970.2	4	<0.001
Is expensive to produce	80%	33%	89%	69%	9%	1883.7	4	<0.001
May contain pathogens, such as bacteria or viruses	26%	54%	44%	70%	73%	591.2	4	<0.001
Is prone to technology failure	49%	46%	82%	73%	12%	1217.7	4	<0.001
May contain industrial chemicals and other man-made chemicals such as solvents	30%	43%	49%	68%	25%	478.6	4	<0.001
May contain trace elements of health concern, such as boron	25%	41%	48%	63%	29%	383.2	4	<0.001
Does not taste good	18%	34%	43%	52%	35%	281.2	4	<0.001
Could be a health concern, for instance if people would drink it	12%	21%	36%	60%	45%	645.0	4	<0.001
May contain substances such as hormones, etc., which can affect human fertility	20%	30%	36%	53%	17%	383.3	4	<0.001
May have a high salt concentration	24%	23%	52%	38%	15%	402.5	4	<0.001
Because the water cycle is closed, it contains human waste	10%	20%	28%	52%	10%	652.9	4	<0.001
Is disgusting	8%	15%	25%	39%	14%	365.7	4	<0.001
Stains the washing	6%	16%	19%	31%	28%	257.5	4	<0.001

**Table 6:** Changes in water images in Australia 2009 to 2012 (desirable attributes).

	Water type	Wave 1	Wave 2	Wave 3	Wave 4	Wave 5
Is safe for human consumption	Bottled water	93%	93%	93%	93%	
	Current tap water	91%	91%	90%	92%	
	Desalinated water	74%	77%	74%	76%	75%
	Recycled water	57%	58%	54%	58%	52%
	Rainwater from own tank			69%	71%	
Looks absolutely clear	Bottled water	93%	94%	94%	93%	
	Current tap water	71%	74%	71%	71%	
	Desalinated water	72%	73%	73%	73%	78%
	Recycled water	64%	64%	63%	62%	68%
	Rainwater from own tank			58%	58%	
Is odourless	Bottled water	87%	87%	87%	84%	
	Current tap water	62%	65%	61%	61%	
	Desalinated water	61%	64%	62%	60%	72%
	Recycled water	54%	57%	54%	54%	63%
	Rainwater from own tank			69%	67%	
Is healthy	Bottled water	85%	82%	82%	80%	
	Current tap water	80%	80%	75%	76%	
	Desalinated water	60%	63%	58%	58%	62%
	Recycled water	47%	50%	44%	47%	45%
	Rainwater from own tank			67%	70%	
Is environmentally responsible	Bottled water	35%	27%	25%	24%	
	Current tap water	67%	71%	64%	66%	
	Desalinated water	62%	60%	56%	56%	55%
	Recycled water	85%	88%	84%	84%	78%
	Rainwater from own tank			92%	91%	
Increases the availability of freshwater	Bottled water	44%	37%	41%	35%	
	Current tap water	37%	37%	38%	34%	
	Desalinated water	81%	81%	79%	81%	77%
	Recycled water	80%	82%	79%	80%	73%
	Rainwater from own tank			83%	83%	
Providers can be trusted to ensure quality is suitable for the intended usage	Bottled water	72%	68%	69%	72%	
	Current tap water	71%	71%	69%	72%	
	Desalinated water	63%	62%	60%	63%	63%
	Recycled water	59%	58%	53%	56%	54%
	Rainwater from own tank			67%	69%	
Creates new jobs	Bottled water	62%	62%	63%	64%	
	Current tap water	30%	30%	34%	32%	
	Desalinated water	87%	90%	90%	90%	84%
	Recycled water	83%	87%	88%	87%	78%
	Rainwater from own tank			35%	36%	
Can save Australia from drought	Bottled water	25%	21%	23%	22%	
	Current tap water	29%	27%	28%	28%	
	Desalinated water	77%	78%	77%	76%	70%
	Recycled water	81%	83%	83%	84%	74%
	Rainwater from own tank			79%	80%	
Reduces the need for water restrictions	Bottled water	26%	21%	23%	27%	
	Current tap water	22%	23%	23%	21%	
	Desalinated water	72%	73%	77%	74%	70%
	Recycled water	79%	80%	83%	83%	74%
	Rainwater from own tank			84%	84%	
Using it reduces the amount of wastewater discharged to the environment	Bottled water	35%	29%	28%	27%	
	Current tap water	36%	37%	32%	35%	
	Desalinated water	52%	48%	43%	46%	40%
	Recycled water	86%	87%	84%	85%	79%
	Rainwater from own tank			68%	69%	
Is the most responsible water source to use from a public health perspective	Bottled water	46%	39%	43%	40%	
	Current tap water	68%	69%	66%	65%	
	Desalinated water	42%	44%	38%	36%	47%
	Recycled water	42%	43%	35%	34%	41%
	Rainwater from own tank			62%	61%	
Is the most environmentally responsible water source to use	Bottled water	20%	16%	13%	14%	
	Current tap water	52%	52%	40%	42%	
	Desalinated water	42%	39%	31%	30%	38%
	Recycled water	72%	74%	54%	53%	64%
	Rainwater from own tank			90%	89%	
Reduces contamination of beaches	Bottled water	26%	23%	24%	21%	
	Current tap water	36%	39%	30%	32%	
	Desalinated water	40%	39%	37%	36%	33%
	Recycled water	64%	65%	63%	63%	52%
	Rainwater from own tank			54%	56%	

**Table 7:** Changes in water images in Australia 2009 to 2012 (undesirable attributes).

	Water type	Wave 1	Wave 2	Wave 3	Wave 4	Wave 5
Is expensive to be delivered to the consumer	Bottled water	88%	90%	90%	90%	
	Current tap water	31%	30%	38%	40%	
	Desalinated water	77%	75%	82%	82%	76%
	Recycled water	54%	53%	63%	62%	56%
	Rainwater from own tank			9%	8%	
Uses a lot of energy in production	Bottled water	70%	74%	77%	75%	
	Current tap water	27%	25%	34%	35%	
	Desalinated water	87%	88%	91%	90%	83%
	Recycled water	64%	64%	72%	72%	52%
	Rainwater from own tank			7%	7%	
Is expensive to produce	Bottled water	78%	81%	80%	82%	
	Current tap water	27%	27%	33%	33%	
	Desalinated water	87%	85%	89%	89%	84%
	Recycled water	62%	60%	69%	68%	55%
	Rainwater from own tank			9%	7%	
May contain pathogens, such as bacteria or viruses	Bottled water	29%	30%	26%	27%	
	Current tap water	55%	55%	54%	55%	
	Desalinated water	50%	46%	44%	45%	40%
	Recycled water	70%	69%	70%	69%	61%
	Rainwater from own tank			73%	68%	
Is prone to technology failure	Bottled water	44%	46%	49%	48%	
	Current tap water	38%	38%	46%	44%	
	Desalinated water	73%	75%	82%	78%	67%
	Recycled water	65%	66%	73%	70%	55%
	Rainwater from own tank			12%	12%	
May contain industrial chemicals and other man-made chemicals such as solvents	Bottled water	28%	30%	30%	32%	
	Current tap water	40%	41%	43%	46%	
	Desalinated water	50%	46%	49%	52%	44%
	Recycled water	67%	65%	68%	70%	61%
	Rainwater from own tank			25%	24%	
May contain trace elements of health concern, such as boron	Bottled water	26%	29%	25%	29%	
	Current tap water	40%	42%	41%	44%	
	Desalinated water	49%	46%	48%	49%	42%
	Recycled water	65%	63%	63%	67%	58%
	Rainwater from own tank			29%	29%	
Does not taste good	Bottled water	19%	18%	18%	17%	
	Current tap water	31%	31%	34%	32%	
	Desalinated water	42%	40%	43%	44%	35%
	Recycled water	49%	50%	52%	53%	45%
	Rainwater from own tank			35%	33%	
Could be a health concern, for instance if people would drink it	Bottled water	14%	14%	12%	13%	
	Current tap water	20%	18%	21%	20%	
	Desalinated water	38%	36%	36%	37%	32%
	Recycled water	59%	57%	60%	58%	56%
	Rainwater from own tank			45%	43%	
May contain substances such as hormones, etc., which can affect human fertility	Bottled water	20%	22%	20%	23%	
	Current tap water	27%	29%	30%	33%	
	Desalinated water	36%	33%	36%	36%	31%
	Recycled water	54%	53%	53%	55%	52%
	Rainwater from own tank			17%	17%	
May have a high salt concentration	Bottled water	23%	23%	24%	24%	
	Current tap water	22%	22%	23%	22%	
	Desalinated water	54%	51%	52%	54%	45%
	Recycled water	38%	38%	38%	38%	29%
	Rainwater from own tank			15%	14%	
Because the water cycle is closed, it contains human waste	Bottled water	13%	11%	10%	11%	
	Current tap water	21%	21%	20%	20%	
	Desalinated water	29%	26%	28%	26%	22%
	Recycled water	51%	49%	52%	51%	46%
	Rainwater from own tank			10%	10%	
Is disgusting	Bottled water	7%	8%	8%	8|%	
	Current tap water	16%	14%	15%	14%	
	Desalinated water	25%	23%	25%	26%	23%
	Recycled water	40%	35%	39%	42%	37%
	Rainwater from own tank			14%	15%	
Stains the washing	Bottled water	7%	5%	6%	5%	
	Current tap water	17%	13%	16%	13%	
	Desalinated water	20%	18%	19%	20%	18%
	Recycled water	28%	29%	31%	30%	26%
	Rainwater from own tank			28%	24%	
