# Supplementary material for: Branding water
Source: Water Res. 2014 Jun 15;57(100):325–38. doi: 10.1016/j.watres.2014.03.056 (PMC4045201; doi:10.1016/j.watres.2014.03.056)
Supplement: Supplementary file 1 [file mmc1.pdf]

QUESTIONNAIRE

Dear Panellist,

This questionnaire is part of an Australian Research Council funded research project conducted by the University of Wollongong and the University of Melbourne. The aim is to better understand environmental attitudes of Australians, particularly with respect to water use.
This is the only way that we can learn how Australians really feel about environmental issues.

The survey will take about 30 minutes to complete and we will credit your account with $4 on completion of this survey.

It is very important that you answer all questions honestly, even if you feel that a different answer would appear to be more socially desirable.

Should you have any concerns or complaints regarding the way in which the research is or has been conducted, please contact the Secretary of the University of Wollongong Human Research Ethics Committee on (02) 4221 4457.

Thank you very much for helping us with our research!

Please click Next to continue to the first question.

How old are you? ……..

Are you…?

Female
Male

Which, if any of the following states or territories do you reside in?

Australian Capital Territory
New South Wales
Northern Territory
Queensland
South Australia
Tasmania
Victoria
Western Australia
Other

Please select the highest level of education you have attained to date:

Postgraduate Degree or equivalent
Doctoral Degree Level
Master Degree Level
Graduate Diploma/Graduate Certificate or equivalent

Graduate Diploma Level
Graduate Certificate Level
Bachelor Degree or equivalent
Advanced Diploma/ Diploma or equivalent
Advanced Diploma and Associate Degree Level
Diploma Level
Certificate Level
Certificate III & IV Level
Certificate I & II Level
Secondary Education
Senior Secondary Education
Junior Secondary Education

Primary Education

Primary Education

Pre- Primary Education

Pre-primary Education

Other Education

Non-award Courses

Miscellaneous Education

The following questions are regarding four different types of water – recycled water, desalinated water, tap water, bottled water and tank water.
Please consider each statement in light of the types of water and state your opinion by either choosing YES or NO. If you are unsure do not worry, your best estimate is fine.

	Recycled water	Desalinated water	Current tap water	Bottled water	Rainwater from own rainwater tank
Is potentially a health risk if I drink it	Yes (1) / No (0)	Yes (1) / No (0)	Yes (1) / No (0)	Yes (1) / No (0)	Yes (1) / No (0)
I know a lot about it	Yes (1) / No (0)	Yes (1) / No (0)	Yes (1) / No (0)	Yes (1) / No (0)	Yes (1) / No (0)
Is of very high quality	Yes (1) / No (0)	Yes (1) / No (0)	Yes (1) / No (0)	Yes (1) / No (0)	Yes (1) / No (0)
I have used it before	Yes (1) / No (0)	Yes (1) / No (0)	Yes (1) / No (0)	Yes (1) / No (0)	Yes (1) / No (0)
Contains chemicals, such as chlorine	Yes (1) / No (0)	Yes (1) / No (0)	Yes (1) / No (0)	Yes (1) / No (0)	Yes (1) / No (0)
May contain purified domestic wastewater	Yes (1) / No (0)	Yes (1) / No (0)	Yes (1) / No (0)	Yes (1) / No (0)	Yes (1) / No (0)
May contain purified industrial wastewater	Yes (1) / No (0)	Yes (1) / No (0)	Yes (1) / No (0)	Yes (1) / No (0)	Yes (1) / No (0)
Producing it could be an environmental concern	Yes (1) / No (0)	Yes (1) / No (0)	Yes (1) / No (0)	Yes (1) / No (0)	Yes (1) / No (0)
Is safe for human consumption	Yes (1) / No (0)	Yes (1) / No (0)	Yes (1) / No (0)	Yes (1) / No (0)	Yes (1) / No (0)
Using it reduces the amount of wastewater discharged to the environment	Yes (1) / No (0)	Yes (1) / No (0)	Yes (1) / No (0)	Yes (1) / No (0)	Yes (1) / No (0)
Could be a health concern, for instance if people would drink it	Yes (1) / No (0)	Yes (1) / No (0)	Yes (1) / No (0)	Yes (1) / No (0)	Yes (1) / No (0)
Quality can be affected by the way it is transported to your home	Yes (1) / No (0)	Yes (1) / No (0)	Yes (1) / No (0)	Yes (1) / No (0)	Yes (1) / No (0)
Is expensive for the consumer	Yes (1) / No (0)	Yes (1) / No (0)	Yes (1) / No (0)	Yes (1) / No (0)	Yes (1) / No (0)

The following questions are regarding four different types of water – recycled water, desalinated water, tap water, bottled water and tank water.
Please consider each statement in light of the types of water and state your opinion by either choosing YES or NO. If you are unsure do not worry, your best estimate is fine.

	Recycled water	Desalinated water	Current tap water	Bottled water	Rainwater from own rainwater tank
May contain pathogens, such as bacteria or viruses	Yes (1) / No (0)	Yes (1) / No (0)	Yes (1) / No (0)	Yes (1) / No (0)	Yes (1) / No (0)
Uses a lot of energy in production	Yes (1) / No (0)	Yes (1) / No (0)	Yes (1) / No (0)	Yes (1) / No (0)	Yes (1) / No (0)
May contain substances such as hormones or pharmaceutically active compounds which can affect human fertility	Yes (1) / No (0)	Yes (1) / No (0)	Yes (1) / No (0)	Yes (1) / No (0)	Yes (1) / No (0)
May contain industrial chemicals and other man-made chemicals such as solvents	Yes (1) / No (0)	Yes (1) / No (0)	Yes (1) / No (0)	Yes (1) / No (0)	Yes (1) / No (0)
May contain trace elements of health concern, such as boron	Yes (1) / No (0)	Yes (1) / No (0)	Yes (1) / No (0)	Yes (1) / No (0)	Yes (1) / No (0)
May have a high salt concentration	Yes (1) / No (0)	Yes (1) / No (0)	Yes (1) / No (0)	Yes (1) / No (0)	Yes (1) / No (0)
Increases the availability of freshwater	Yes (1) / No (0)	Yes (1) / No (0)	Yes (1) / No (0)	Yes (1) / No (0)	Yes (1) / No (0)
Can save Australia from drought	Yes (1) / No (0)	Yes (1) / No (0)	Yes (1) / No (0)	Yes (1) / No (0)	Yes (1) / No (0)

Is expensive to produce	Yes (1) / No (0)	Yes (1) / No (0)	Yes (1) / No (0)	Yes (1) / No (0)	Yes (1) / No (0)

The following questions are regarding four different types of water – recycled water, desalinated water, tap water, bottled water and tank water.

Please consider each statement in light of the types of water and state your opinion by either choosing YES or NO. If you are unsure do not worry, your best estimate is fine.

	Recycled water	Desalinated water	Current tap water	Bottled water	Rainwater from own rainwater tank
Is expensive to be delivered to the consumer	Yes (1) / No (0)	Yes (1) / No (0)	Yes (1) / No (0)	Yes (1) / No (0)	Yes (1) / No (0)
Reduces the need for water restrictions	Yes (1) / No (0)	Yes (1) / No (0)	Yes (1) / No (0)	Yes (1) / No (0)	Yes (1) / No (0)
Does not taste good	Yes (1) / No (0)	Yes (1) / No (0)	Yes (1) / No (0)	Yes (1) / No (0)	Yes (1) / No (0)
Requires chemicals to be produced	Yes (1) / No (0)	Yes (1) / No (0)	Yes (1) / No (0)	Yes (1) / No (0)	Yes (1) / No (0)
Produces greenhouse emissions	Yes (1) / No (0)	Yes (1) / No (0)	Yes (1) / No (0)	Yes (1) / No (0)	Yes (1) / No (0)
Is environmentally responsible	Yes (1) / No (0)	Yes (1) / No (0)	Yes (1) / No (0)	Yes (1) / No (0)	Yes (1) / No (0)
Is odourless	Yes (1) / No (0)	Yes (1) / No (0)	Yes (1) / No (0)	Yes (1) / No (0)	Yes (1) / No (0)
Is the most environmentally responsible water source to use	Yes (1) / No (0)	Yes (1) / No (0)	Yes (1) / No (0)	Yes (1) / No (0)	Yes (1) / No (0)
Is healthy	Yes (1) / No (0)	Yes (1) / No (0)	Yes (1) / No (0)	Yes (1) / No (0)	Yes (1) / No (0)

The following questions are regarding four different types of water – recycled water, desalinated water, tap water, bottled water and tank water.

Please consider each statement in light of the types of water and state your opinion by either choosing YES or NO. If you are unsure do not worry, your best estimate is fine.

	Recycled water	Desalinated water	Current tap water	Bottled water	Rainwater from own rainwater tank
Is the most responsible water source to use from a public health perspective	Yes (1) / No (0)	Yes (1) / No (0)	Yes (1) / No (0)	Yes (1) / No (0)	Yes (1) / No (0)
Is prone to technology failure	Yes (1) / No (0)	Yes (1) / No (0)	Yes (1) / No (0)	Yes (1) / No (0)	Yes (1) / No (0)
Because the water cycle is closed, it contains human waste	Yes (1) / No (0)	Yes (1) / No (0)	Yes (1) / No (0)	Yes (1) / No (0)	Yes (1) / No (0)
Looks absolutely clear	Yes (1) / No (0)	Yes (1) / No (0)	Yes (1) / No (0)	Yes (1) / No (0)	Yes (1) / No (0)
Providers of the water source can be trusted to ensure quality is suitable for the intended usage	Yes (1) / No (0)	Yes (1) / No (0)	Yes (1) / No (0)	Yes (1) / No (0)	Yes (1) / No (0)
Stains the washing	Yes (1) / No (0)	Yes (1) / No (0)	Yes (1) / No (0)	Yes (1) / No (0)	Yes (1) / No (0)
Is disgusting	Yes (1) / No (0)	Yes (1) / No (0)	Yes (1) / No (0)	Yes (1) / No (0)	Yes (1) / No (0)
Creates new jobs	Yes (1) / No (0)	Yes (1) / No (0)	Yes (1) / No (0)	Yes (1) / No (0)	Yes (1) / No (0)
Reduces contamination of beaches	Yes (1) / No (0)	Yes (1) / No (0)	Yes (1) / No (0)	Yes (1) / No (0)	Yes (1) / No (0)

For the following questions
we will use the term “**recycled water”** to describe **“highly purified wastewater”.**
we will use the term **“desalinated water”** to describe **“highly purified seawater”**
and we will use the term “rainwater” to describe rainwater from a rainwater collection tank on your property (rainwater collected from the roof of your house)
We will also assume that both recycled and desalinated water were treated to the same level of water quality.
Please click “Next” to continue.

Have you ever used recycled water?	□ Yes	□ No	□ Not sure
Have you ever used desalinated water?	□ Yes	□ No	□ Not sure
Have you ever used rainwater?	□ Yes	□ No	□ Not sure

The following section seeks your opinion with regards to RECYCLED WATER. Please answer the following section with RECYCLED WATER in mind.

For the following question, imagine that you live in a town where:

- **Dams supplying household water currently hold 20 % of capacity**
- **Level 5 Mandatory Water Restrictions are in place for the use of tap water (no outside watering of gardens, no watering systems, no refilling swimming pools, no washing vehicles except for windows and headlights)**
- **Recycled water is readily available without restrictions**

Under these circumstances, please indicate how likely you would be to use RECYCLED WATER for the following purposes by placing the slider in the respective position along the line.

Some of these behaviors may not apply to you, e.g. because you do not have a swimming pool. In this case please tick the “not applicable” option.

Please "left-click" to activate the slider button. A change in color from lighter to darker will indicate that the slider button is now activated. Drag towards and release the slider button to the point on the slider which expresses your opinion.

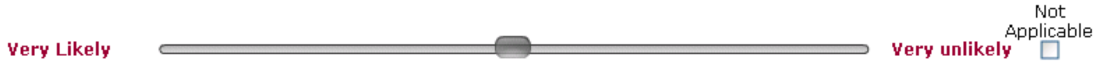

Watering the garden (flowers, trees, shrubs)
Washing clothes, doing laundry
Cooking
Showering / taking a bath
Drinking
Brushing teeth
Bathing the baby
Filling up the fish pond or aquarium
Toilet flushing
Cleaning the house, windows, driveways
Watering of garden – vegetables, herbs to be eaten raw
Washing the car
Refilling / topping up the swimming pool
Feeding my pets

The following section seeks your opinion with regards to DESALINATED WATER. Please answer the following section with DESALINATED WATER in mind.

Again, please imagine that you live in a town where:

- **Dams supplying household water currently hold 20 % of capacity**
- **Level 5 Mandatory Water Restrictions are in place for the use of tap water (no outside watering of gardens, no watering systems, no refilling swimming pools, no washing vehicles except for windows and headlights)**
- **Desalinated water is readily available without restrictions**

Under these circumstances, please indicate how likely you would be to use DESALINATED WATER for the following purposes by placing the slider in the respective position along the line.

Some of these behaviors may not apply to you, e.g. because you do not have a swimming pool. In this case please tick the “not applicable” option.

Please "left-click" to activate the slider button. A change in color from lighter to darker will indicate that the slider button is now activated. Drag towards and release the slider button to the point on the slider which expresses your opinion.

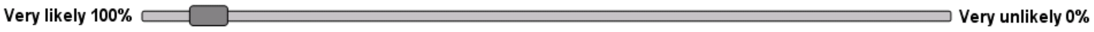

Watering the garden (flowers, trees, shrubs)
Washing clothes, doing laundry
Cooking
Showering / taking a bath
Drinking
Brushing teeth
Bathing the baby
Filling up the fish pond or aquarium
Toilet flushing
Cleaning the house, windows, driveways
Watering of garden – vegetables, herbs to be eaten raw
Washing the car
Refilling / topping up the swimming pool
Feeding my pets

The following section seeks your opinion with regards to RAINWATER FROM YOUR OWN RAINWATER TANK. Please answer the following section with TANK WATER in mind.

Again, please imagine that you live in a town where:

- **Dams supplying household water currently hold 20 % of capacity**
- **Level 5 Mandatory Water Restrictions are in place for the use of tap water (no outside watering of gardens, no watering systems, no refilling swimming pools, no washing vehicles except for windows and headlights)**
- **Rainwater from a rainwater tank on your property is readily available without restrictions**

Under these circumstances, please indicate how likely you would be to use RAINWATER FROM YOUR OWN RAINWATER TANK (if you do not have one, please imagine you do) for the following purposes by placing the slider in the respective position along the line.

Some of these behaviors may not apply to you, e.g. because you do not have a swimming pool. In this case please tick the “not applicable” option.

Please "left-click" to activate the slider button. A change in color from lighter to darker will indicate that the slider button is now activated. Drag towards and release the slider button to the point on the slider which expresses your opinion.

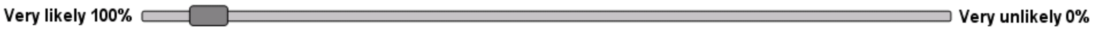

Watering the garden (flowers, trees, shrubs)
Washing clothes, doing laundry
Cooking
Showering / taking a bath
Drinking
Brushing teeth
Bathing the baby
Filling up the fish pond or aquarium
Toilet flushing
Cleaning the house, windows, driveways
Watering of garden – vegetables, herbs to be eaten raw
Washing the car
Refilling / topping up the swimming pool
Feeding my pets

Please rank the following five kinds of water with respect to [DRINKING/SHOWERING/WATERING YOUR VEGETABLE GARDEN/WASHING YOUR CAR].
Please assign a 1 for the water you would use the most for the purpose named above, a 2 for the water you would use 2nd most for the purpose named above…and a 5 for the water you would use the least.

Bottled water
Current tap water
Recycled water
Desalinated water
Rainwater from your own rainwater tank

Are you currently connected to a main (centralised) water supply system e.g. water provided to a city or town by a water supply authority?

Yes
No

Do you currently have a rainwater tank installed at your home?
Yes
No

What size is it?
Less than 2,000L
2,000-3,999L
4,000-6,999L
7,000 or more

In what year did you install your tank?
2010
2009
2008
2007
2006
2005 or earlier

Did you receive a government rebate for your rainwater tank?
Yes
No

What kind of rebate was it?
Select all that are applicable
federal government
state government
local government
other (please specify_____________)
not sure

What was the MAIN reason you installed your rainwater tank?
To reduce my water bill
To reduce the impact of water restrictions on my household activities
Because of the rebate
To help avoid the need for new large-scale water sources to be constructed (dams, desalination plants, recycling plants)
To reduce my reliance on mains water supply
For environmental reasons
Other (please specify_______)

Where do you use your rainwater?
Outdoor only
Outdoor and indoor

For what purposes do you use your rainwater?
Watering my garden (not edible plants)
Watering my garden (edible plants)
Washing my car/boat
Washing my pavers/driveway
Other (please specify)

For what purposes do you use your rainwater?
Watering my garden (not edible plants)
Watering my garden (edible plants)
Washing my car/boat
Washing my pavers/driveway
Toilet
Washing machine
Shower
Kitchen tap
Other (please specify)

<u>IF OUTDOOR only</u>
Why don't you have your rainwater tank connected to your indoor plumbing?
Costs too much to connect the plumbing
Was not aware this was possible
Don't want to use rainwater for these purposes
Too much effort
Other (please specify)

IF NOT
Why not?

Too expensive
Not enough space
Not interested
Other

Are you aware that state, federal, and some local governments provide financial rebates up to $1500 for rainwater tank installation?

Yes
No

If the government introduced a scheme where they provided you with a water tank for free, but you had to pay the installation costs (e.g. cement block, water pump, plumbing connection to toilet/laundry), would you participate in this scheme?

Yes
No

If the scheme gave you a free tank AND covered the cost of installation, but you still had to pay for the plumbing to connect the tank for indoor uses, would you participate and pay the additional money for the plumbing?

Yes
No

If the scheme provided a free tank, covered the cost of installation AND the cost of connection for indoor uses, would you participate in this scheme?

Yes
No

Imagine you had a rainwater tank. Would you still want to purchase water from your water authority (e.g. for drinking etc.)?
You have a rainwater tank. Do you still purchase water from your water authority (e.g. for drinking etc.)?

Yes
No

Given that you would, in this situation, use a lot less water supplied to you by your water authority, would you be willing to pay a higher price for this water?
Given that you have to purchase less water (because you have a rainwater tank), would you be willing to pay a higher price for this water?

Yes
No

How much of a price increase, per kL of water, would you be willing to accept in the above situation? 0-100% _______%

Have you seen or heard any advertising campaigns about water conservation?

Yes
No

Where did you see or hear these advertising campaigns about water conservation? *Select as many as apply*

Radio
Television
Print Media
From the water authority with my bill
Other (please specify___________)

Have you seen or heard any advertising campaigns about recycled water?

Yes
No

Where did you see or hear these advertising campaigns about recycled water? *Select as many as apply*

Radio
Television
Print Media
From the water authority with my bill
Other (please specify___________)

Have you seen or heard any advertising campaigns about desalinated water?

Yes
No

Where did you see or hear these advertising campaigns about desalinated water?
Select as many as apply

Radio
Television
Print Media
From the water authority with my bill
Other (please specify___________)

Have you seen or heard any advertising campaigns about rainwater tanks?

Yes
No

Where did you see or hear these advertising campaigns about rainwater tanks?
Select as many as apply

Radio
Television
Print Media
From the water authority with my bill
Other (please specify___________)

In your opinion, have any advertisements or campaigns influenced your decision to adopt water conservation practices within and around your home?

Yes
No
Unsure

Are you aware of any water wise rebates that are currently offered by government bodies?

Yes
No
No, but I would like to find out more about them and how I can make use of them in my home

Where did you hear about water conservation rebates that you may be entitled to?
Select as many as apply

Exhibition stand
Poster/billboard
Television
Word of mouth
Friends and family
Radio
Newspaper
Conference
From the water authority
On the internet

Please specify which water conservation rebates you applied for within your home:

Select as many as apply

Rainwater Tank Rebate (outdoor)
Rainwater Tank Rebate (indoor and outdoor)
Washing Machine Rebate
Do-It-Yourself Water Saving Kits
Toilet Replacement Rebate
Showerhead Rebate
Other, please specify:______________________________
None

Do you trust your local water authority to deliver safe drinking water?

Yes, I trust my local water authority a 100%
Yes, I generally trust my local water authority
No, I have some concerns
No, I do not trust my local water authority at all

To conclude the survey we would like to ask you a few questions about yourself:

What is your annual household income?

Under $20,000
$21,000 to $40,000
$41,000 to $60,000
$61,000 to $80,000
$81,000 to $100,000
Over $100,000
Would rather not say

How large is the town / city you live in?
If you are not sure your best guess is fine.

0-5,000
5,001-20,000

20,001-50,000
50,001-100,000
100,001-250,000
250,001-500,000
500,001-1,000,000
1,000,001-2,000,000
2,000,001-3,000,000
3,000,001-4,000,000
Greater than 4,000,000

How strong is your feeling of belonging and attachment to the region you live in?

Strong
Moderate
Weak
Non existent

Please complete the sentence by ticking one of the answers below: Would you ……

1. Prefer to stay in the region?
2. Prefer to move out of the region but stay in the country?
3. Prefer to move abroad?
4. Or do you not care where you live?

What is your ancestry?
You may choose more than one.

Aboriginal
Australian
Other Oceanian
North West European
South East European
North African and Middle Eastern
South East Asian (e.g. Vietmanese, Filipino, Indonesian)
North East Asian (e.g. Chinese)
Southern and Central Asian (e.g. Indian)
North American
South American
African
Other
Prefer not to say

Do you speak a language other than English at home?

No, English Only
Yes, Arabic (including Lebanese)
Yes, Australian Indigenous Languages
Yes, Cantonese
Yes, Croatian
Yes, Dutch
Yes, French
Yes, German
Yes, Greek
Yes, Hebrew
Yes, Hindi
Yes, Hungarian
Yes, Indonesian
Yes, Italian
Yes, Japanese
Yes, Korean
Yes, Macedonian
Yes, Malay
Yes, Maltese
Yes, Mandarin
Yes, Polish

Yes, Portuguese
Yes, Russian
Yes, Serbian
Yes, Spanish
Yes, Tagalog (Filipino)
Yes, Thai
Yes, Turkish
Yes, Vietnamese
Yes, Auslan (Australian Sign Language)
Yes, Other

How Australian do you feel?

0%
10%
20%
30%
40%
50%
60%
70%
80%
90%
100%

Have you ever experienced water restrictions? Yes No

To which extent have you had to change your behavior because of water restrictions?

Not at all
Slightly
Strongly

To which extent do you feel limited by water restrictions?

Not at all
Slightly
Strongly

Is religion important in your life?

Yes
No
I am not sure
I would rather not say

On how many days a week do you usually read the news and current affairs section of the newspaper?
Please only count the days on which you read the news and current affairs sections and exclude days on which you only read other sections (e.g. sports, entertainment, etc.).

1
2
3
4
5
6
7
I read the newspaper on an irregular basis (less than once a week)
I don't read the newspaper at all

What is your favorite newspaper?

- The Australian
- The Financial Review
- The Canberra Times
- The Daily Telegraph
- Sydney Morning Herald
- The Age
- The Herald Sun
- The Courier-Mail
- The Advertiser
- The West Australian
- The Mercury
- The N.T. News
- A regional daily newspaper
- A local daily newspaper
- Other paper

On how many days a week do you usually watch news and current affairs programs on TV?
Please only count the days on which you watch news and current affairs programs and exclude days on which you watch dramas, sports, etc. only.

- 1
- 2
- 3
- 4
- 5
- 6
- 7
- I watch TV news on an irregular basis (less than once a week)
- I don't watch TV news at all

What is your favourite television channel?

- ABC1
- ABC2
- Seven
- Nine
- TEN
- ONE
- SBS ONE
- SBS TWO
- Another channel not listed, which I receive free-to-air
- Another channel not listed, which I receive from a subscription TV service

On how many days a week do you usually listen to news programs on the radio?
Please only count the days on which you listen to news on the radio.

- 1
- 2
- 3
- 4
- 5
- 6
- 7
- I listen to the radio news on an irregular basis
(less than once a week)
- I do not listen to the radio news at all

Thank you very much for your help!
